# The effectiveness and safety of acupuncture therapy for Guillain–Barré syndrome: A systematic review and meta-analysis protocol

**DOI:** 10.1097/MD.0000000000018619

**Published:** 2020-01-10

**Authors:** Zhu Fan, Biyuan Liu, Yili Zhang, Man Li, Tao Lu

**Affiliations:** a School of Life Sciences; b School of Traditional Chinese Medicine, Beijing University of Chinese Medicine, Beijing, China; c School of Medicine, University of St Andrews, Scotland, UK.

**Keywords:** acupuncture, Guillain–Barré syndrome, randomized controlled trials, systematic review, protocol

## Abstract

**Background:**

Guillain–Barré syndrome (GBS) is the most common acute paralytic neuropathy. Many clinical trials indicate acupuncture provides a good effect as a complementary therapy of Western medicine for GBS. The objective of this systematic review protocol is to provide the evidence to evaluate the effectiveness and safety of acupuncture on the treatment of GBS.

**Methods:**

We will search relevant randomized controlled trials investigating the effect of acupuncture for GBS in following databases from start to October 2019: PubMed, Embase, the Cochrane Library, CINAHL Complete, National Digital Science Library, China National Knowledge Infrastructure, and Wanfang Database without language restriction. For articles that meet our inclusion criteria, 2 researchers will extract the data information independently, and assess the risk of bias and trial quality by the Cochrane collaboration's tool. All data will be analyzed by RevMan V.5.3.3 statistical software.

**Results:**

According to the Barthel index of Activities of Daily Living (ADL) and the Medical Research Council (MRC) muscle scale, the efficacy and safety of acupuncture for GBS will be determined in this study.

**Conclusion:**

This systemic review will provide high quality evidence to judging whether acupuncture provides benefits to treat GBS.

Prospero registration number: CRD42019158710.

## 1 Introduction

Guillain–Barré syndrome (GBS) is an immune-mediated, acute polyradiculoneuropathy disease,^[1]^ with GBS incidence about 0.6 to 3 per 100,000 person-years in worldwide.^[2]^ According to the epidemiologic survey, the risk of GBS increases with age, and the prevalence of GBS in men is higher than that in women.^[3]^ The typical clinical features in GBS is symmetrical paralysis of limbs, which usually starts from the lower limbs and gradually spreads to the upper limbs and face,^[4,5]^ even can lead to respiratory paralysis and endanger life. Usually, the severity of symptoms peaked in 2 to 4 weeks and gradually recovered in the following months or years.

The GBS mainly caused by virus infection of peripheral nerve or nerve root, and cause extensive inflammatory demyelination of peripheral nervous system. About 75% of patients with GBS have previous infection history within 6 weeks before onset, usually respiratory infection and gastrointestinal infection.^[6]^ GBS could be divided into 4 subphenotypes, including acute inflammatory demyelinating polyradiculoneuropathy (AIDP), Miller Fisher syndrome (MFS), acute motor axonal neuropathy (AMAN), and acute motor-sensory axonal neuropathy, the most common subphenotypes of GBS are AIDP and AMAN.^[7]^

There is no specific drug for GBS, the treat strategies of GBS include general medical care and immunologic treatment.^[8,9]^ Though the treatment of plasma exchange (PE) and intravenous immunoglobulin (IVIg) are shown effectively in hastening recovery and improving outcome,^[10–12]^ many patients still occur several residual adverse reactions, like fatigue, pain, anxiety, and disease recurrence.^[13–16]^ With the increasing demand of patients for quality of life, it presents a new challenge to the treatment strategy of GBS.

Acupuncture, as a traditional Chinese treatment, has been widely used in cancer and neurovascular diseases as a complementary treatment, and has excellent effects on pain alleviate, anxiety relief, limb function recovery, and other discomfort symptoms.^[17–19]^ In addition, there are many reports about acupuncture treatment of GBS, mostly in Chinese.

According to our preliminary search, we found that acupuncture treatment and acupuncture combined with other methods to treat GBS are gradually increasing; however, there is no systematic review and meta-analysis of acupuncture treatment GBS. The review aims to evaluate the effectiveness and safety of acupuncture as a clinical complementary treatment for GBS.

## 2 Material and methods

This protocol has been registered at PROSPERO (registration number: CRD42019158710). The preferred reporting items for the systematic review and meta-analysis (PRISMA) will be followed in this study.^[20]^

### 2.1 Inclusion criteria

#### 2.1.1 Type of studies

Only relevant randomized controlled trials (RCTs) which explore the efficacy and safety of acupuncture in the treatment of GBS will be included. Comments, case report, quasi-RCT, animal experiments, or non-RCTs will be excluded.

#### 2.1.2 Types of participants

Participants who have been diagnosed with GBS according to diagnostic criteria (Asbury 1990)^[21]^ will be included in this review. There will be no restriction on their age, sex, and race.

#### 2.1.3 Types of interventions

We will only include studies which interventions involved acupuncture alone (including needle acupuncture, electroacupuncture, warm acupuncture) or combined with any other western medicine treatments or immune preparation.

#### 2.1.4 Types of comparisons

The following treatment will be control interventions: immune preparation; western medicine including oral drugs, external drugs, and injections.

#### 2.1.5 Types of outcomes

##### 2.1.5.1 Primary outcomes

The primary outcome measurement will be an improvement of the Barthel index of ADL and the MRC muscle scale. According to the Barthel index score, the ability of daily living activities can be divided into 3 levels: good, medium, and poor, which are >60 points into good; 60 to 41 points into medium, if <40 points, it is poor, means severe dysfunction, and most daily life activities cannot be completed independently. The MRC muscle scale classifies muscle strength into 13 levels for evaluation.

##### 2.1.5.2 Secondary outcomes

The secondary outcomes of the review will include as followed: satisfaction rates, quality of life, and adverse events.

### 2.2 Search strategy

We will search relevant RCTs in following databases from start to October 2019: PubMed, Embase, the Cochrane Library, CINAHL Complete, National Digital Science Library, China National Knowledge Infrastructure, and Wan-fang Database without language restriction. Our search strategy includes main keywords such as “acupuncture,” “acupoint,” “needling,” “body acupuncture,” “electroacupuncture,” “scalp acupuncture,” “ear acupuncture,” “skin acupuncture,” “warm needling,” “Guillain–Barré syndrome,” “randomized controlled trials,” and “GBS,” respectively, for literature search in Chinese and English. The search strategy for PubMed is shown in Table 1.

**Table 1:**
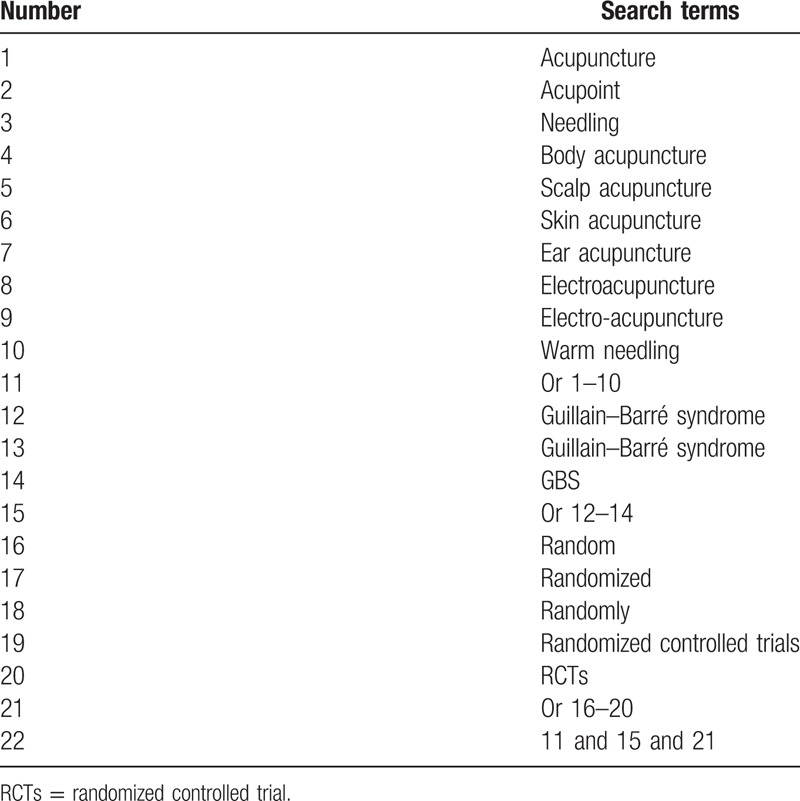
Search strategy for PubMed.

### 2.3 Selection of studies

**Figure 1:**
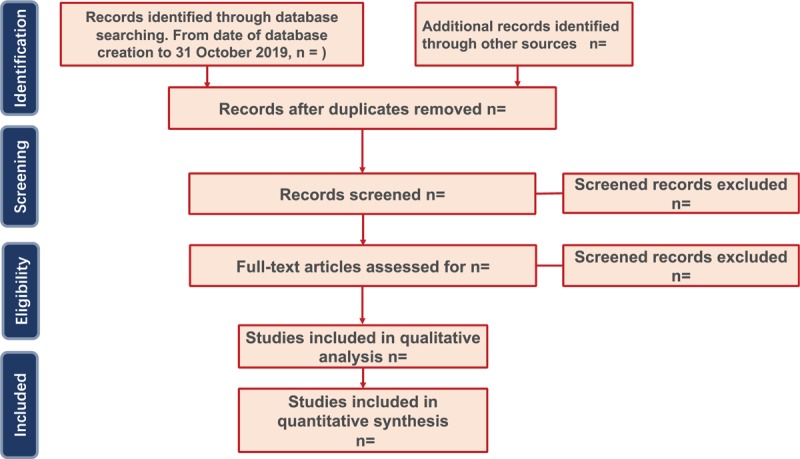
Flow diagram of study selection process.

We will use EndNote X9 software to manage results which extract from the above electronic databases. Two reviewers (FZ and LBY) will review the titles and abstracts of each record independently to exclude articles that do not meet our inclusion criteria. Based on the preliminary research results, a full-text investigation will be examined based on the inclusion criteria. If there is a disagreement between the 2 researchers, it will be solved by the discussion with the third reviewer (ZYL). The details of all study selection process are shown in the flowchart (Fig. 1).

### 2.4 Data extraction

According to the inclusion, we will create a standard data extraction form containing specified outcomes before data extraction. For studies meeting the inclusion criteria, 2 researchers (FZ and LBY) will extract data from them independently and fill them into a standard extraction form. Following aspects will be included in this form: general information (author, country, year of publication), study design (random method, blinding of participants), characteristics of participants, interventions in control group and treatment group (acupoint selection, treatment frequency, and duration), and outcomes. One of the researchers will contact the authors to get the complete data in case the data are incomplete. If there is a disagreement between the 2 researchers, it will be solved by the discussion with the 3rd reviewer (ZYL).

### 2.5 Assessment of risk of bias

Two independent reviewers (FZ and LBY) assess the risk of bias of each included article according following characteristics (Cochrane risk of bias tool): random sequence generation, allocation concealment, blinding, completeness of outcome data, selective outcome reporting, and other bias. The risks will be classified into 3 levels: low, high, and unclear. Any disagreements will be resolved through discussion, or if agreement cannot be reached, a 3rd reviewer (ZYL) will be consulted.

### 2.6 Data analysis

We use the review manager software v.5.3.3 provided by Cochrane Collaboration for statistical analysis. For continuous data, we use mean difference or standardized mean difference for analysis, and for dichotomous data analysis, we calculate it by risk ratio. The fixed-effects models were used to evaluate data with homogeneous data (*I*^2^ < 50%). When *I*^2^ ≥ 50%, which was regarded as substantial statistical heterogeneity, we use the random-effects model. If the number of included studies is <2 or heterogeneity is apparent, the results of our systematic review will be narratively reported.

### 2.7 Analysis of subgroups

If sufficient data are available, we will conduct a subgroup analysis of 3 subtypes of GBS, including AIDP, axonal forms of the disease, and MFS, so as to evaluate the difference in the therapeutic effect of acupuncture on different subtypes.

### 2.8 Sensitivity analysis

If there are sufficient data which available to analyze, we will conduct sensitivity analysis on the primary outcomes to test the robustness of the review conclusions, including the quality of the methods, the quality of the studies, and the impact of sample size and missing data.

### 2.9 Ethics and dissemination

As there is a systematic review of the effectiveness and safety of acupuncture in the treatment of GBS, we do not involve animals and individual experiments, so ethical approval will not be required. The results will be published in peer-reviewed journals once our analysis is completed

## 3 Discussion

The GBS is a serious threat to the quality of life and physical and mental health of patients. Although treatment of PE and IVIg effectively reduce the mortality and related complications, it also puts forward new requirements for the treatment of GBS. How to make patients with GBS to reduce pain, recover exercise ability as early as possible, enhance muscle strength, improve psychologic state, and return to the society and family as soon as possible is an urgent problem to be solved. According to the latest review and clinical research, as a traditional Chinese medicine therapy, acupuncture can reduce pain, increase muscle strength, improve neurologic function, and improve patients’ psychologic state.^[17,22–25]^

However, there is no complete evaluation of the clinical evidence of acupuncture treatment for GBS up to now. Therefore, we intend to make a systematic review and meta-analysis to evaluate the efficacy and safety of acupuncture in the treatment of GBS. We hope that the review can provide more evidence and help clinicians to provide more diversified options in the treatment of GBS. In addition, the review may have limitations, the results may be affected by the quality of Chinese and English articles, and it is difficult to carry out single blind or double-blind experimental measures in acupuncture treatment.

## Author contributions

**Data curation:** Zhu Fan, Biyuan Liu, Yili Zhang.

**Formal analysis:** Yili Zhang, Man Li.

**Methodology:** Zhu Fan, Biyuan Liu.

**Project administration:** Tao Lu.

**Software:** Yili Zhang

**Validation:** Man Li

**Writing – original draft:** Zhu Fan, Biyuan Liu

**Writing – review & editing:** Yili Zhang.

Tao Lu orcid: 0000-0002-8247-8387.
